# Cross-domain transfer learning strategy enhances interpretability of deep learning model explanations

**DOI:** 10.1038/s41598-026-59076-8

**Published:** 2026-06-24

**Authors:** Matteo Zannini, Alexander Hammer, Hagen Malberg, Martin Schmidt

**Affiliations:** Institute of Biomedical Engineering, TU Dresden, Dresden, Germany

**Keywords:** Computational biology and bioinformatics, Engineering, Mathematics and computing

## Abstract

Clinical decision-making increasingly relies on deep neural networks (DNNs), yet their deployment in practice requires transparent and interpretable predictions. Explainable artificial intelligence (xAI) methods can identify input regions relevant to a model’s decision, but their clinical interpretability remains limited. In this study, we investigated whether inductive transfer learning (TL) can reinforce domain-specific feature separation in xECGArch, a two-branch convolutional neural network for atrial fibrillation (AF) detection from electrocardiograms (ECGs). Each branch was pre-trained on a task aligned with its designated feature domain, P wave detection for morphology and RR interval variability prediction for rhythm, then fine-tuned on binary AF classification using an iterative layer freezing schedule. Deep Taylor decomposition (DTD) was applied to analyze explanations across all configurations. Fine-tuning accuracy ranged from 85.70% to 95.23%, remaining comparable to the original xECGArch architecture and previous TL-based approaches. However, DTD analysis demonstrated that morphology pre-training directed relevance toward P waves, whereas rhythm pre-training concentrated explanations on R peaks, with domain specificity increasing as more layers were frozen. These findings suggest that inductive TL can encourage domain-specific feature attribution in DNNs, improving the alignment of post-hoc explanations with clinically meaningful ECG regions.

## Introduction

The integration of deep learning (DL) algorithms into clinical decision support systems holds great promise for improving diagnostic accuracy and efficiency. However, for clinicians to trust such systems, their decisions must be transparent, allowing validation against medical knowledge^1^.

In the context of classification of electrocardiograms (ECGs), deep neural networks (DNNs) have demonstrated high accuracy in the detection of multiple cardiac arrhythmias^2–9^, and in particular of atrial fibrillation (AF), the most prevalent one^10^. AF is characterized on the ECG by the absence of P waves, their replacement by fibrillatory waves, and an irregular rhythm, known as absolute arrhythmia^11^. A major obstacle in the clinical application of DL algorithms is their intrinsic lack of explainability, which is vital to increase user trust^1^. Explainable AI (xAI) enables post-hoc analysis of model decision-making by mapping input samples to classification outcomes^12^. xAI methods can be used to highlight ECG segments that are relevant to the classification. However, the resulting sample-wise relevance attributions often lack clear causal interpretation, limiting their clinical utility^13^. While concept-based explanation strategies can address this, they rely on prior domain knowledge and predefined concepts. An alternative approach is to incorporate interpretability directly into the model architecture^14^. By dimensioning the receptive field size, networks can be guided to autonomously learn distinct features. An example of this is xECGArch^15^, a DL architecture that leverages receptive field constraints to separately learn rhythmic and morphological features from ECGs for reliable AF detection. The model architecture consists of two independent convolutional neural networks (CNNs), the long-term model (LTM) and the short-term model (STM). Systematic validation^16,17^ of explanations extracted with deep Taylor decomposition (DTD)^18^ demonstrated that the STM self-learns morphological features of the ECG, namely fibrillatory waves and P waves, while the LTM focuses on QRS complexes, with relevance changes correlating with rhythm^17^. However, the learned morphological and rhythmic features may overlap in some cases. For example, the QRS complex morphology may influence the decision-making of the LTM instead of the rhythmic spacing between R peaks, while rhythmic information embedded in beat morphology, such as the QT interval, might play a role in the decision process of the STM. From a clinical perspective, such feature entanglement reduces the correspondence between the model explanations and the reasoning process used by cardiologists, who typically distinguish rhythm-related and morphology-related ECG abnormalities separately during diagnosis. Consequently, DTD relevance may be attributed to signal regions that are not directly associated with the intended feature domain of the model, limiting the interpretability and reliability of the explanations as clinical decision-support evidence. Therefore, a method capable of enforcing a clearer separation between rhythm-specific and morphology-specific feature learning is required.

Transfer learning (TL) is a machine learning (ML) technique that allows the application of knowledge from a previously trained DL model to a new task and domain. Given a source domain $D_s$, a target domain $D_t$ and their respective learning tasks $T_s$ and $T_t$, TL uses the knowledge in $D_s$ and $T_s$ to improve the learning of the target predictive function $f_t(.)$ in the target domain $D_t$^19^. In supervised learning problems, a commonly employed variant is inductive TL, in which $T_s$ is different from $T_t$, while $D_s$ and $D_t$ can be related or not^20^. Network-based deep TL refers to reusing the partial DNN that was pre-trained on $D_s$, including its structure and weights, and transferring it to be a part of the network to be used on $D_t$ to perform the target task $T_t$^21^. The network weights can be frozen, making them unable to be updated during the training process. Deciding whether to freeze or fine-tune layers depends on the distribution of the available data and on the source and target tasks^20^.

In studies on ECG analysis, TL was often implemented with the aim of increasing the classification performance and robustness of DL models or reducing the computational cost of fine-tuning^22–31^. Chon et al.^32^ and Avetisyan et al.^33^ improved the classification performance of multiple arrhythmias by pre-training their models on larger datasets. The effects of different pre-training tasks on the target task were investigated in multiple studies^34–38^. In particular, Nguyen et al.^38^ developed a measure to estimate the transferability in multi-label ECG diagnosis. TL for domain adaptation in AF detection was explored by Yu et al.^39^ and Argha et al.^40^, while other studies^41–43^ employed unsupervised pre-training to address the issue of scarce annotations. These studies showed that TL may increase the capability of DNNs to generalize across different contexts, often improving the performance of the model on a new task or dataset. However, the domain shift addressed in these works was predominantly task-to-task or dataset-to-dataset: models were typically adapted from multi-pathology classifiers to single-condition detection, or from larger datasets to a new one. Moreover, xAI techniques have only been implemented in two studies involving TL for ECG analysis^44,45^. The use of TL to enforce domain-specific feature separation within a multi-branch architecture may therefore provide a strategy to improve the clinical plausibility of xAI explanations, but this possibility has not yet been systematically investigated.

**Fig. 1:**
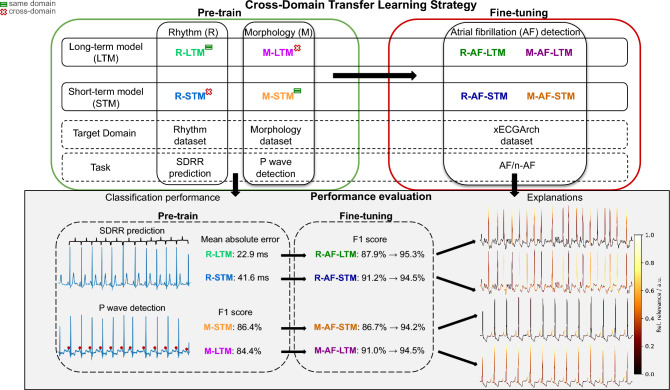
Schematic representation of the proposed transfer learning strategy with cross-learning for improved domain specificity of model explanations. The long-term model (LTM) is initially trained to predict the standard deviation of successive RR intervals (SDRR) using the ECG samples of the rhythm dataset, while the short-term model (STM) is trained to detect P waves using the samples of the morphology dataset. The pre-training tasks are switched (cross-domain task), leading to a total of four pre-trained models: the rhythm-LTM (R-LTM), the morphology-STM (M-STM), the morphology-LTM (M-LTM) and the rhythm-STM (R-STM). Subsequently, the models are fine-tuned on the xECGArch dataset to classify AF and n-AF ECGs. To determine the effects of pre-training on the downstream task, the classification performance of each model is assessed and explanations are derived using explainable AI. In the lower panel, explanations of AF examples are shown for the rhythm-pre-trained models to illustrate relevance attribution to R peaks during irregular rhythm, whereas n-AF examples are shown for the morphology-pre-trained models to illustrate relevance attribution to P waves.

In this study, we define improved interpretability as a stronger alignment between DTD relevance attributions and pre-specified, clinically meaningful ECG regions associated with the intended feature domain of each model. To overcome the limitations of xECGArch, we developed a TL pipeline aimed at enforcing a clearer separation between morphological and rhythmic feature learning, by directing the attention of each network exclusively toward its target feature domain. We pre-trained the STM and LTM on designated morphology-related and rhythm-related tasks and subsequently fine-tuned the networks on AF detection. The chosen rhythmic task was prediction of the standard deviation of the RR intervals (SDRR) to characterize absolute arrhythmia. On the other hand, detection of P waves was an appropriate choice as a morphological task since the absence of P waves, replaced by fibrillatory waves, is a fundamental marker of AF. The STM and LTM were modified into the morphology-STM (M-STM) and rhythm-LTM (R-LTM) and pre-trained on their respective main tasks, the STM on the detection of P waves and the LTM on SDRR prediction. The models were then modified into morphology-LTM (M-LTM) and rhythm-STM (R-STM) and pre-trained on the complementary tasks, which are referred to as cross-tasks. Next, we fine-tuned the pre-trained models to classify ECGs into AF and non-AF. During fine-tuning, the model layers were iteratively frozen to retain the knowledge acquired during pre-training. Finally, we systematically analyzed the relevance of the features during classification using DTD and appropriate statistical tools. A schematic representation of our pipeline is shown in Fig. 1. We expected the models pre-trained on the main tasks to exclusively focus on their target feature domains, namely the M-AF-STM to focus on the morphology of the signals in terms of the presence of P waves or fibrillatory waves, and the R-AF-LTM to prioritize the irregular occurrence of the QRS complex. Moreover, we hypothesized that freezing more layers would reinforce such focus, leading to an increase in interpretability as defined in this study. On the other hand, we expected the models pre-trained on the cross-tasks, the R-AF-STM and M-AF-LTM, to show more heterogeneous explanations, with overlapping focus on morphological and rhythmic features.

## Results

### Network performance

We pre-trained the long-term and short-term branches of xECGArch on two occasions, once on SDRR prediction and once on P wave detection. For these tasks, two separate databases were employed, the rhythm dataset and the morphology dataset. The rhythm dataset contained a total of 19,047 10-second Einthoven lead II ECGs from four publicly available datasets, namely PTB-XL^46,47^, Georgia-12-Lead^48^, China Physiological Signal Challenge 2018^49^ and Chapman-Shaoxing^50^. In each ECG, the R peaks were annotated and the SDRR was extracted. The morphology dataset contained 2,716 10-second Einthoven lead II ECGs from the MIT-BIH Arrhythmia Database (MITDB)^51,52^, the Lobachevsky University Electrocardiography Database^53^ and the Brno University of Technology ECG Signal Database (BUTDB)^54^, each containing P wave annotations. The performance results of each network during pre-training are shown in Table 1.

We optimized the R-LTM and the R-STM in a 5-fold cross-validation grid search monitoring the cross-validation mean absolute error (MAE$_{\text {cv}}$), given as the average MAE over the folds. On the validation folds, the optimized R-LTM predicted the SDRR with an average MAE of 18.29 ms, whereas the R-STM obtained an MAE of 41.20 ms. On the test set, the best R-LTM obtained a MAE of 22.92 ms and the R-STM a MAE of 41.60 ms. The optimal hyperparameters for both networks were a batch size (bs) of 8 and a starting learning rate (lr) of 0.0001.

The STM and LTM were then modified into the morphology short-term model (M-STM) and morphology long-term model (M-LTM) and trained to detect P waves. The networks were optimized in a 5-fold cross-validation grid search monitoring the cross-validation F1 score (F1$_{\text {cv}}$), given as the mean F1 score minus one standard deviation over the folds. The M-STM obtained the highest F1$_{\text {cv}}$ of 76.07% using a starting lr of 0.0001 and bs of 8, compared to the F1$_{\text {cv}}$ of 73.18% reached by the M-LTM with an optimal lr of 0.0001 and bs of 4. On the test subset, the M-STM achieved a binary F1 score of 86.42% compared with 84.37% obtained by the M-LTM. When increasing the tolerance window for detection to 5 samples, the M-STM achieved an F1 score of 91.24%, and the M-LTM reached an F1 score of 90.73%.

Subsequently, the models were initialized with the pre-trained weights and fine-tuned to classify the signals of the xECGArch dataset into AF and non-AF. The dataset contained a total of 9,854 10-second Einthoven lead II ECGs from the same four datasets as the rhythm dataset, each labeled as AF or non-AF. The fine-tuning results are illustrated in Table 2.

**Table 1:** Pre-training results of each model during cross-validation (cv) and on the test subset. The F1$_{\text {cv}}$ was computed as the mean F1 score across the five folds minus one standard deviation, and the MAE$_{\text {cv}}$ was computed as the average MAE across the five folds. Acc: accuracy, bs: batch size, F1: F1 score, lr: learning rate, MAE: mean absolute error, Prec: precision, Sens: sensitivity, Spec: specificity, Val.: validation.

Model	bs	lr	Val. MAE$_{\mathrm{cv}}$ [ms]	Test MAE [ms]	Model	bs	lr	Val. F1$_{\mathrm{cv}}$ [%]	Test Prec/Sens/Acc/Spec/F1 [%]
**R-LTM**	8	0.0001	18.29	22.92	**M-LTM**	4	0.0001	73.18	83.04/85.74/99.98/99.87/84.37
**R-STM**	8	0.0001	41.20	41.60	**M-STM**	8	0.0001	76.07	86.22/86.61/99.95/99.97/86.42
**Target domain**	Rhythm dataset				Morphology dataset				
**Task**	SDRR prediction				P wave detection				

Table 2 shows the fine-tuning performance across configurations of frozen layers (fl). Subtable 2a summarizes the scores of the R-AF-LTM. On the validation folds, the highest F1$_{\textit{cv}}$ of 95.08% was obtained without fl using a bs of 8 and a lr of 0.001, whereas the highest test performance was achieved with 3 fl (*accuracy* = 95.23%, *F1 score* = 95.28%), slightly outperforming the original LTM (*accuracy* = 95.00%, *F1 score* = 95.13%). Performance decreased markedly in the 8-fl configuration (*accuracy* = 87.02%, *F1 score* = 87.92%). Subtable 2b presents the performance of the M-AF-LTM. The highest F1$_{\textit{cv}}$ of 93.29% was obtained with 3 fl, using a bs of 8 and lr of 0.001, whereas the best test performance was obtained without fl (*accuracy* = 94.22%, *F1 score* = 94.45%). The 8-fl configuration achieved the lowest test performance (*accuracy* = 90.67%, *F1 score* = 90.98%). Subtable 2c shows the results of the R-AF-STM. The highest validation performance was obtained without fl (*F1*$_{\textit{cv}}$ = 93.36%; bs = 16, lr = 0.001), while the best test performance was reached with 4 fl (*accuracy* = 94.32%, *F1 score* = 94.51%). Freezing more than 5 layers reduced performance, with the lowest scores obtained in the 8-fl configuration (*accuracy* = 90.97%, *F1 score* = 91.20%). Finally, Subtable 2d summarizes the performance of the M-AF-STM. The highest F1$_{\textit{cv}}$ of 92.83% was achieved on the validation folds by the model without fl, using a bs of 8 and a lr of 0.001. This configuration also produced the highest test performance (*accuracy* = 93.92%, *F1 score* = 94.18%), nearly matching the original STM (*accuracy* = 94.01%, *F1 score* = 94.18%). Overall, increasing the number of fl reduced classification performance across all models, particularly in the most constrained configurations.

**Table 2:**
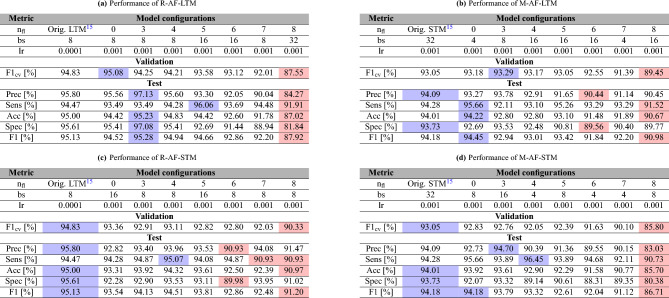
Fine-tuning performance of each model during cross-validation (cv) and on the test subset. The F1$_{\text {cv}}$ was computed as the mean F1 score across the five folds minus one standard deviation. For each metric, the best score across configurations is highlighted in blue and the worst in red. Acc: accuracy, bs: batch size, F1: F1 score, fl: frozen layers, lr: learning rate, n$_{\text {fl}}$: number of frozen layers, Prec: precision, Sens: sensitivity, Spec: specificity.

### Model explanations

Using DTD, we extracted explanations of each trained model in terms of the relative relevance (rR) of individual samples for the classification decision and visualised them as heatmaps overlaid on the signals.

**Fig. 2:**
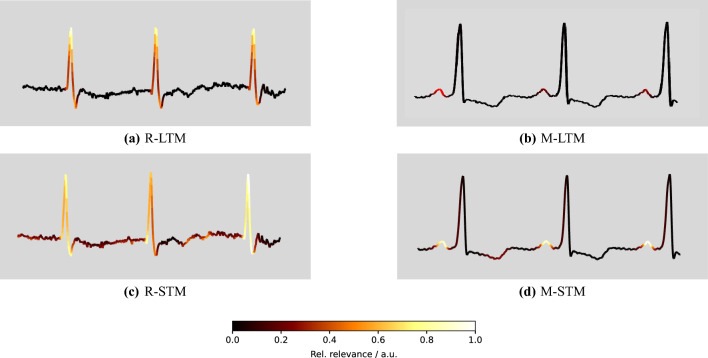
Explanations by deep Taylor decomposition (DTD)^18^ of exemplary classified ECG segments of the rhythm dataset (**a**, **c**) and morphology dataset (**b**, **d**) by the pre-trained models. The colorized ECG segments display the relative relevance attributed by DTD after pre-training.

In Fig. 2, exemplary pre-training ECGs from the rhythm and morphology datasets are displayed. The color intensity encodes the samples’ rR according to DTD, normalized between 0 and 1. According to DTD, the R-LTM (subfigure 2a) focused exclusively on the QRS complex, with the highest rR on the R peaks, whereas the M-LTM (subfigure 2b) assigned the highest relevance to the P waves. The explanations of the R-STM (subfigure 2c) highlighted the entire QRS complex, indicating a broader relevance distribution than observed for the R-LTM. The explanations of the M-STM (subfigure 2d) emphasized the P waves, with a more pronounced relevance attribution than observed for the M-LTM.

**Fig. 3:**
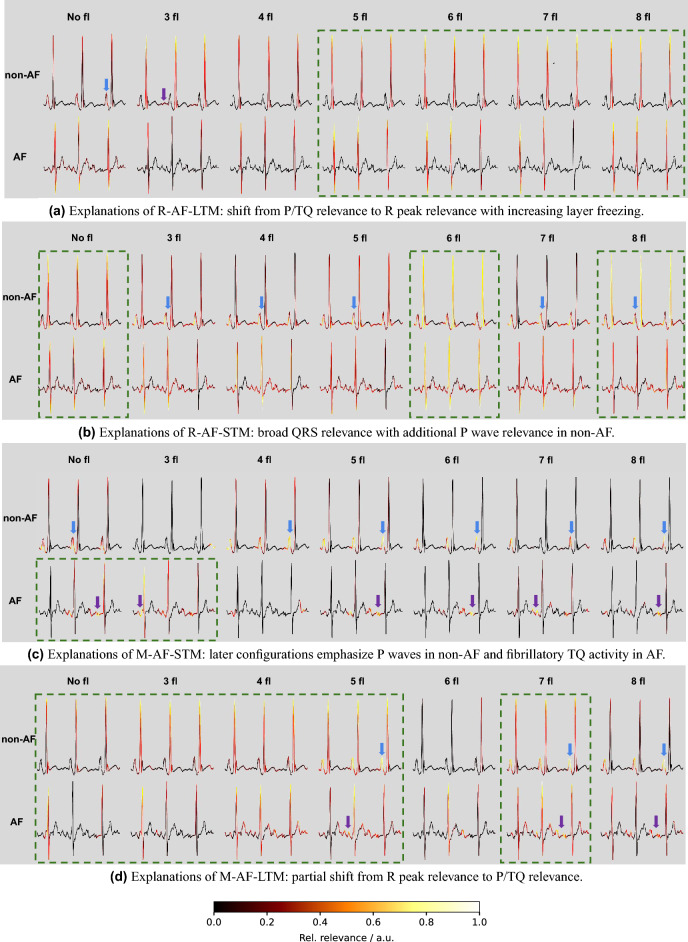
Explanations by deep Taylor decomposition^18^ of the fine-tuned models for exemplary classified ECG segments of the xECGArch dataset^15^. The colorized ECG segments display the relative relevance after fine-tuning with increasing numbers of frozen layers (fl) in non-atrial fibrillation (AF) and AF samples from the test set. Blue arrows indicate P wave relevance, purple arrows indicate TQ interval relevance, and green boxes indicate QRS complex relevance.

Figure 3 shows exemplary ECG segments from the xECGArch dataset correctly classified as non-AF and as AF. In the R-AF-LTM examples (Fig. 3a), increasing the number of fl shifted the explanations by DTD from partial emphasis on the P wave and TQ interval in initial configurations (no fl and 3 fl), toward the QRS complex in later configurations, with increasing emphasis on the R peaks in both AF and non-AF. In contrast, the R-AF-STM (Fig. 3b) showed more heterogeneous relevance patterns, with relevance distributed across QRS-related regions and, in several non-AF configurations, along the left flank of the P wave (configurations with 3, 4, 5, and 7 fl). The explanations of the M-AF-STM (Fig. 3c) partially emphasized the QRS complex in AF in initial configurations (no fl and 3 fl), whereas P waves became increasingly highlighted in non-AF and fibrillatory-wave regions in AF, particularly in later configurations. The M-AF-LTM (Fig. 3d) displayed a less consistent pattern with relevance especially assigned to R peaks in most configurations. Further examples are shown in Supplementary Figure S1.

### Relation between model explanations and ECG intervals

**Fig. 4:**
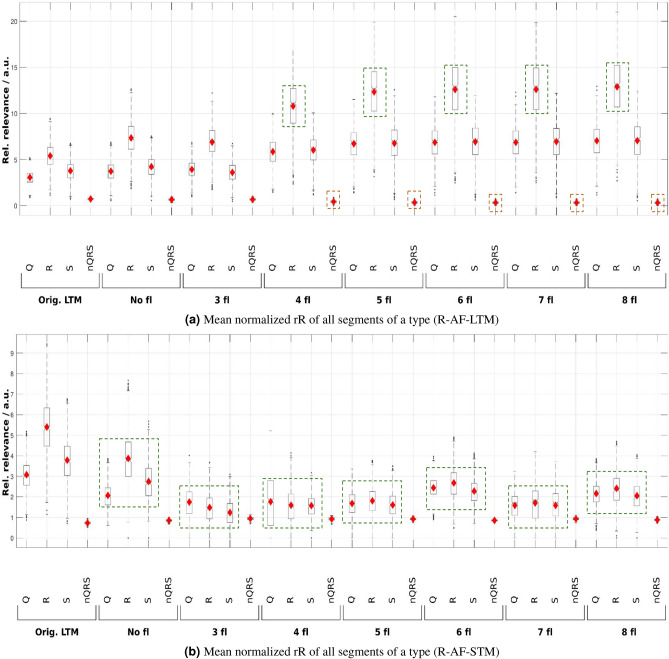

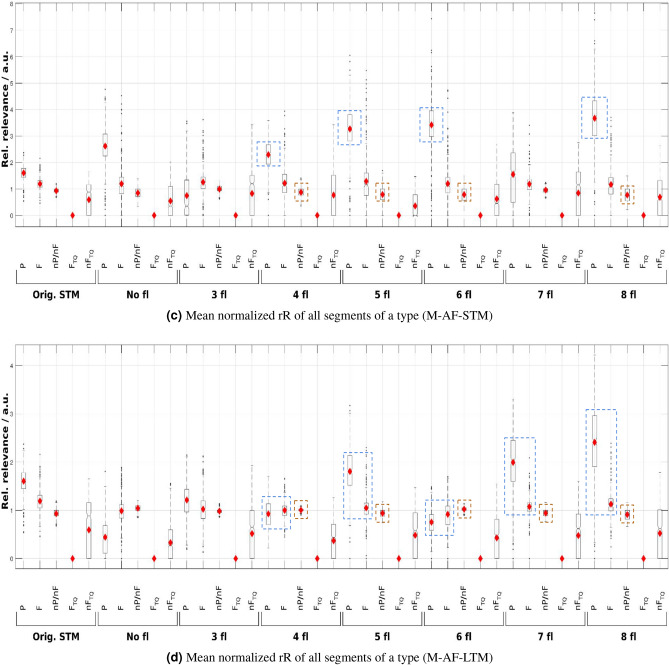
Distribution of intervals’ mean relative relevance for classification by each model configuration of frozen layers (fl). The boxplot limits represent the upper and lower quartiles, with the notch in between representing the median and the red diamond representing the mean. The whiskers are limited to 1.5-times the interquartile range and outliers are shown as dots. Dashed boxes indicate ECG intervals with dominant relative relevance in representative configurations, whereas the annotations summarize the main explanation trends across increasing numbers of fl. Statistical comparisons between intervals’ mean relative relevance in AF and non-AF by Tukey-Kramer post-hoc test are shown in Table 3. The explanations of the R-AF-LTM (a) showed increasing relevance of R peaks and decreasing relevance of nQRS with more fl, whereas the explanations of the R-AF-STM (b) showed broad relevance across the QRS complex without clear R peak specialization. The M-AF-STM (c) attributed increasing relevance to the P waves and decreasing relevance to nP/nF intervals in later configurations, while a partial increase in P and F wave relevance relative to nP/nF regions was observed in later configurations of the M-AF-LTM (d). F: fibrillatory waves, F$_{\text {TQ}}$: Fibrillatory waves within the TQ interval, nF$_{\text {TQ}}$: TQ interval without fibrillatory waves, nP/nF: all intervals excluding P and fibrillatory waves, nQRS: all intervals excluding the QRS complex, P: P waves, Q: Q waves, R: R peaks, S: S waves.

We systematically analyzed the explanations of each model to investigate the relation between rR and diagnostically relevant beat segments. We chose Q, R, S, and notQRS (nQRS) segments for the analysis of the models that were pre-trained on the rhythmic task, and P wave, F waves, and regions excluding P and F waves (nP/nF), TQ interval without F waves (nF$_{\text {TQ}}$), and F waves within the TQ interval (F$_{\text {TQ}}$) for the models pre-trained on morphology. We determined the mean rR of each beat segment type within each recording. The descriptive statistics of the mean rR across intervals and model configurations are presented as box plots in Fig. 4. Furthermore, we assessed statistical significance using a 2-factor analysis of variance (ANOVA) and post-hoc analysis using Student’s *t*-tests with Tukey-Kramer alpha-error correction and Cohen’s *d*^55^ for effect sizes. Table 3 reports the results of post-hoc analysis for rR differences between R intervals across configurations of the R-AF-LTM and R-AF-STM and between P and F intervals across configurations of the M-AF-STM and M-AF-LTM.

In the explanations of the R-AF-LTM (subfigure 4a), the R peak was the most relevant segment across fl configurations and progressively increased in relevance with more fl, rising from a median normalized rR of 5.35 in the original model to 13.12 in the 8-fl configuration (+145%). At the same time, relevance assigned to nQRS regions decreased by up to 56% in AF explanations. Most consecutive transitions from 0 to 5 fl produced significant changes in R peak relevance ($p < 0.0001$, $|d| \ge 0.8$), whereas later transitions between 6, 7, and 8 fl were not significantly different (Table 3). These findings suggest progressive specialization toward rhythm-related ECG features. In contrast, the R-AF-STM (subfigure 4b) showed more heterogeneous relevance distributions across the QRS complex. Although most transitions between configurations were statistically significant ($p < 0.001$ to $p < 0.0001$), R peak relevance did not consistently increase relative to the original model and reached its minimum in the 3-fl configuration (−73%). These results suggest that the R-AF-STM did not develop the same progressive R peak specialization observed in the R-AF-LTM. The explanations of the M-AF-STM (subfigure 4c) suggested an increased focus on morphology-related intervals. In non-AF explanations, P waves became the dominant interval in most configurations, with median rR increases up to +128% relative to the original model. In AF explanations, fibrillatory TQ activity remained relatively stable across configurations, whereas the TQ interval without fibrillatory waves received minimal attribution. Most configuration transitions produced highly significant changes in P/F-wave-related relevance ($p < 0.0001$), with predominantly large effect sizes in non-AF explanations (Table 3). The M-AF-LTM (subfigure 4d) showed a weaker but still observable shift toward morphology-related intervals. P wave relevance increased in later configurations, reaching a maximum increase of +50.42% in the 8-fl configuration, while fibrillatory TQ activity became more prominent in the 5-, 7-, and 8-fl configurations. However, the interval specialization remained less consistent than in the M-AF-STM, particularly in AF explanations.

As a robustness analysis, we additionally fitted linear mixed-effects models using recording as a random intercept. Interval-level models were fitted to the target-domain intervals only, R peaks for the rhythm-pre-trained models and P and F wave regions for the morphology-pre-trained models, whereas recording-level models included all interval categories. Across all four models, fl configuration remained highly significant in the interval-level analyses, and the recording-level models showed significant effects of interval, configuration, and their interaction (*p* < 0.0001 for all tests). These findings indicate that the observed relevance differences remained significant after accounting for recording- and patient-level clustering. The complete results of the mixed-effects analyses are provided in the Supplementary Material.

**Table 3:** Results of the Tukey-Kramer post-hoc test on differences in R peaks or P and F waves for increasing amounts of frozen layers (n$_{\text {fl}}$) regarding their relative relevance, including significance values (–: not significant, *: $p < 0.05$, **: $p < 0.001$, ***: $p < 0.0001$) and Cohen’s *d*^55^ for effect sizes (†: $0.2 \le |d| < 0.5$, ††: $0.5 \le |d| < 0.8$, †††: $0.8 \le |d|$).

Target interval	R-AF-LTM		M-AF-LTM		R-AF-STM		M-AF-STM	
	R peak		P/F wave		R peak		P/F wave	
n$_{\text {fl}}$	AF	nAF	AF	nAF	AF	nAF	AF	nAF
Orig. vs 0	*** †††	*** †††	*** †††	*** †††	*** †††	*** †††	*** †††	*** †††
0 vs 3	*	***	***	*** †††	*** †††	*** †††	***	*** †††
3 vs 4	*** †††	*** †††	***	*** †††	–	–	***	*** †††
4 vs 5	*** †††	*** †††	***	*** †††	–	*** †††	***	*** †††
5 vs 6	–	–	*** †††	*** †††	*** ††	*** †††	*** †	*** †††
6 vs 7	–	–	*** †††	*** †††	** ††	*** †††	*** †	*** †††
7 vs 8	–	–	*** †††	*** †††	*** ††	*** †††	*** †	*** †††

**Fig. 5:**
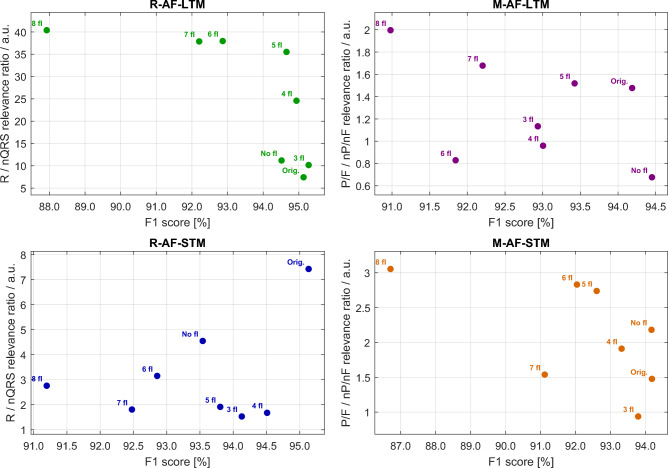
Performance–interpretability trade-off across model configurations. For rhythm-pre-trained models, the domain specificity ratio was defined as R peak/nQRS relevance. For morphology-pre-trained models, the ratio was defined as P/F wave/nP/nF relevance. The ratio was averaged across AF and non-AF recordings and plotted against the corresponding F1 score. fl: frozen layers, Orig: original model.

We additionally calculated a compact domain specificity ratio for each configuration. For R-AF-LTM and R-AF-STM, this ratio was defined as the mean rR assigned to the R peak divided by the mean rR assigned to nQRS regions. For M-AF-LTM and M-AF-STM, it was defined as the mean rR assigned to P/F wave regions divided by the mean rR assigned to nP/nF regions. The ratio was averaged across AF and non-AF recordings and plotted against the corresponding F1 score to visualize the trade-off between classification performance and domain-specific relevance, as shown in Figure 5. The ratio was intended as an exploratory summary of explanation alignment for relative comparison across configurations. No formal optimization criterion was applied to jointly maximize classification performance and domain specificity.

The trade-off analysis showed that configurations with more fl generally increased domain specificity, particularly for the R-AF-LTM and M-AF-STM, but this was accompanied by reduced F1 scores in the most constrained configurations. For the R-AF-LTM, the domain specificity ratio increased from 7.43 in the original configuration to 40.39 in the 8-fl configuration. Similarly, the M-AF-STM increased from a ratio of 0.91 in the configuration with 3 fl to 3.05 in the last configuration. In contrast, the R-AF-STM showed reduced domain specificity compared with the original configuration, ranging from 1.53 to 4.55, while the M-AF-LTM showed more moderate changes, ranging from 0.68 to 2.00.

## Discussion

The features of AF can be partitioned into two domains, a rhythmic and a morphological one. Cardiologists assess the presence of AF based on such distinct feature domains: the absence of P waves and the presence of fibrillatory waves represent morphological indicators, while the irregular timing of heartbeats constitutes the rhythmic hallmark. The architecture of xECGArch reflects this clinical reasoning by assigning the STM to morphological features and the LTM to rhythmic features. However, the learned representations may overlap across the two branches, potentially undermining the interpretability of model explanations. If the ECG segments highlighted by xAI methods cannot be clearly attributed to either the morphological or the rhythmic domain, clinicians cannot reliably trace the model’s reasoning back to established diagnostic criteria, limiting its utility as a decision-support tool.

Separating the feature domains more rigorously is therefore essential to produce explanations that more closely align with clinically meaningful ECG regions and with how cardiologists interpret ECGs. To this end, we applied an inductive, network-based TL pipeline to xECGArch. While the architecture is already designed to learn rhythmic and morphological features separately, TL offers a mechanism to actively reinforce this separation: by pre-training each branch exclusively on tasks from its designated feature domain and subsequently freezing layers during fine-tuning, we expected the networks to retain domain-specific representations and leverage them for AF classification. This approach aimed not only to preserve or improve classification performance, but primarily to reduce the overlap of highlighted signal segments in post-hoc explanations, thereby enhancing domain specificity.

Moreover, we hypothesized that pre-training each model on its designated task would lead to a clearer separation of explanations when compared to pre-training on the cross-tasks, providing further evidence that the TL pipeline successfully enforces domain-specific feature learning. In a preparation study^56^, we pre-trained the LTM of xECGArch to detect R peaks and fine-tuned it on AF detection by iteratively freezing the network layers, which led to an increased focus on the R peaks as more model layers were frozen. In the current study, we investigated the impact of TL on the performance and domain specificity of both branches of xECGArch.

SDRR prediction was selected as the rhythmic pre-training task as it is a commonly adopted measure of heart rate (HR) variability. As highlighted by DTD, the R-LTM (Fig. 2a) and R-STM (Fig. 2c) focused mainly on the QRS complex, and in particular on the R peak. The lower MAE produced by the R-LTM, shown in Table 1, can be traced to its larger receptive field size, which enables the network to focus on a broader region of the input data, likely leading to a more efficient extraction of the SDRR in comparison to the R-STM. As illustrated visually in Figure 3a and quantitatively in Figure 4a, the fine-tuning explanations of the R-AF-LTM showed that DTD predominantly highlighted the QRS complex, with increasing focus on the R peak as more layers were frozen. The limited highlighting of nQRS segments further suggests an exclusive focus on the rhythmic occurrence of the QRS complex. This increase in focus was not equally observed in the explanations of the R-AF-STM (Figures 3b and 4b), where the R peak was consistently highlighted by the model without fl, while the explanations of the configurations with 3, 4, 5, and 7 fl often emphasized the P waves in non-AF, and the R peak and S wave were mostly highlighted in AF, likely suggesting a focus on both morphological and rhythmic features. These results are consistent with our hypothesis that the relevance patterns of the R-AF-LTM are predominantly associated with rhythmic information during classification, while the R-AF-STM displayed more heterogeneous relevance patterns involving both rhythmic and morphological features.

The M-STM and M-LTM showed comparable results in the detection of P waves, as shown in Table 1, with a slight increase in F1 score observed in the predictions of the M-STM. According to DTD, the M-STM showed a strong focus on the P waves during pre-training, as shown in Figure 2d. Interestingly, this was not the case for the explanations of the M-LTM, where the P wave was less highlighted and often the main focus was on the R peaks (Figure 2b). The explanations of the exemplary segments in Figure 3c and the boxplots in Figure 4c displayed an emphasis of the M-AF-STM with fewer fl on multiple segments of the ECG, including the TQ interval and the QRS complex. This suggests either a focus of the model on the whole morphology of the signal, including the amplitude of the QRS complex, or a simultaneous focus on the rhythmic occurrence of the QRS complex. As the amount of fl increased, the explanations of the M-AF-STM increasingly emphasized the occurrence of P waves in non-AF signals and of fibrillatory waves in AF. The systematic comparison of rR across intervals and model configurations, summarized in Table 3, further highlighted significant differences between each fine-tuned model and the original STM, with strong effect sizes for most comparisons in non-AF signals. In AF, the comparisons across configurations displayed weak effect sizes, as the increase in mean rR of F waves was not pronounced. This was observed in the explanations of the M-AF-LTM as well (Figures 3d and 4d), which showed comparatively larger effect sizes in the mean rR of P waves than F waves. In both M-AF-STM and M-AF-LTM classifications, only a few regions of the signal were highlighted, leading to low within-signal rR values. The reduced highlighting of F waves might have also resulted from the limited proportion of AF samples within the pre-training data (see Table 6). Additional mixed-effects analyses using recording-level random intercepts yielded conclusions consistent with the primary ANOVA-based analyses. As each test-set recording corresponded to a unique patient, the random effects also accounted for patient-level clustering.

As displayed in Table 4, the fine-tuning accuracy and F1 score obtained by each model employed in this study were comparable to the original xECGArch scores^15^ and to results achieved in previous studies implementing TL for arrhythmia classification^29,39,42^. Moreover, pre-training on SDRR prediction resulted in an increase of 2% in accuracy and F1 score compared with the model pre-trained on R peak detection in our previous attempt^56^, as well as an increased focus on the R peak for the last configuration of the model.

The limited performance benefit of TL relative to the original model can be attributed to the substantial dissimilarity between the source tasks $T_{s}$ and the target task $T_{t}$. While SDRR prediction and P wave detection both operate on ECG signals, they represent fundamentally different objectives compared to AF classification: one is a regression task targeting beat-to-beat variability, the other a localized morphological detection task. It has been shown that the transferability of learned features decreases as the distance between source and target tasks increases^57^, and that dissimilar source-target configurations can limit the performance gains typically expected from TL^19^. Consequently, the primary value of the proposed TL pipeline lies not in boosting classification performance, but in its capacity to enforce domain-specific feature learning and thereby enhance the domain specificity of model explanations.

**Table 4:** Performance comparison of proposed approaches and previous state of the art. Acc: accuracy, F1: F1 score, Orig: original.

Model	R-AF-LTM	M-AF-STM	R-AF-STM	M-AF-LTM	Orig. LTM^15^	Orig. STM^15^	R-AF-LTM^56^	Montenegro et al.^29^	Xu et al.^39^	Nguyen et al.^42^
Acc [%]	95.23	93.92	94.32	94.22	95.00	94.01	93.20	94.40	97.58	100.00
F1 [%]	95.28	94.18	94.13	94.45	95.13	94.18	93.30	79.72	96.83	97.07

As shown in Table 2 and Figure 5, increasing the number of fl generally reduced classification performance while simultaneously increasing the domain specificity of explanations, particularly for the R-AF-LTM and M-AF-STM. The proposed domain specificity ratio quantitatively captured this trade-off by relating relevance assigned to clinically meaningful ECG regions (R peaks or P/F wave regions) to relevance assigned outside the intended feature domain. Because the ratio was derived from normalized relevance values and no formal uncertainty estimation or denominator stabilization procedures were applied, it should be interpreted as an exploratory summary measure rather than as a definitive metric of interpretability. While the configurations without fl and with 3 and 4 fl showed similar classification outcomes, freezing more layers resulted in a marked decrease in accuracy and F1 score. This was especially evident in the shift from 7 to 8 fl for the main-task models. The R-AF-LTM showed the strongest increase in domain specificity, with the R/nQRS ratio increasing from 7.43 in the original configuration to 40.39 in the 8-fl configuration, while the M-AF-STM showed a comparable increase in the P/F-to-nP/nF ratio from 0.91 to 3.05. However, these highest ratios were associated with reduced classification performance in the most constrained configurations. The mean absolute decrease in F1 score across configurations amounted to 1.10% for the R-AF-LTM and 1.25% for the M-AF-STM, while the M-AF-LTM and R-AF-STM performed more stably across configurations, with average consecutive mean absolute F1 score decreases of 0.37% and 0.58%, respectively. Visual inspection of Figure 5 suggested that intermediate configurations of the main-task models, particularly the 4- and 5-fl configurations of the R-AF-LTM and M-AF-STM, provided an empirically favorable compromise between classification performance and explanation alignment. These configurations should therefore be interpreted as illustrative examples of the observed trade-off rather than as optimal solutions. As an exploratory post-hoc analysis, we additionally ensembled the prediction probabilities of all R-AF-LTM and M-AF-STM configurations using equal weighting. The best-performing combination, R-AF-LTM with 5 fl and M-AF-STM with 4 fl, achieved 96.04% accuracy and 96.20% F1 score, exceeding the original xECGArch ensemble performance reported by Göttling et al.^15^ (*F1 score* = 95.43%, *accuracy* = 95.33%). The next-best combinations also remained above the original ensemble F1 score, suggesting that rhythm- and morphology-specialized models may provide complementary classification evidence. These results support the potential value of ensemble-based extensions, although the analysis should be interpreted as exploratory.

To investigate the effects of RR variability, class label, and R peak amplitude on the certainty of prediction, a generalized linear model (GLM) was calculated with certainty of correct classifications as dependent factor and coefficient of variation of RR intervals CV$_{\text {RR}}$, coefficient of variation of R peak amplitude CV$_{\text {R}_{\text {amp}}}$, label, and the interaction CV$_{\text {RR}}$ ${\times }$label as independent factors. The full results are reported in Table 5. CV$_{\text {R}_{\text {amp}}}$ was never a significant predictor across any model or configuration, with the exception of the R-AF-LTM without fl ($p{<}0.05$), with 3 fl ($p{<}0.05$), and with 5 fl ($p{<}0.0001$). The interaction term CV$_{\text {RR}}{\times }$ was the most consistent predictor across all models and configurations ($p{<}0.0001$ in most cases), indicating that RR variability affects classification certainty differently in AF and non-AF signals throughout, regardless of the number of fl. The only exceptions were the 8-fl configurations of the M-AF-LTM and M-AF-STM, and the 5-fl configuration of the M-AF-STM, where the interaction term was non-significant. The label predictor was significant across most configurations ($p{<}0.05$ to $p{<}0.0001$), reflecting a systematic difference in classification certainty between AF and non-AF signals. Exceptions were observed in the 8-fl configuration of the R-AF-STM and in several configurations of the M-AF-STM (3, 4, and 7 fl), suggesting that in these cases the model did not consistently distinguish between classes in terms of prediction certainty. The R-AF-LTM showed the strongest and most consistent dependence on CV$_{\text {RR}}$ across all configurations ($p{<}0.0001$), while this effect was weaker and configuration-dependent in the remaining models. Notably, the effect of CV$_{\text {RR}}$ was not significant in the last configurations of the M-AF-STM and M-AF-LTM, suggesting minor emphasis on RR variability as freezing increased. The 8-fl configuration of the R-AF-LTM showed particularly impaired classification of non-AF signals (misclassification rate of 18.2%), with misclassified ECGs exhibiting a mean CV$_{\text {RR}}$ of 0.506 compared to 0.229 in correctly classified recordings, suggesting that the model explanations became strongly concentrated on RR spacing as the primary classification cue when fewer layers were trainable. The results of the GLM reinforce the observed outcomes of the systematic analysis by ANOVA (see Figure 4). In particular, the significance of CV$_{\text {RR}}$ and CV$_{\text {RR}}{\times }$label further suggests that the R-AF-LTM with 8 fl focused on the spacing between the QRS complexes to classify the signals, leading to misclassifications when the rhythm was slightly irregular and to an abrupt decrease in detection accuracy. Moreover, RR variability had a marginal effect on the predictions of the M-AF-STM, and it was not a decisive predictor for misclassification for the M-AF-STM with 8 fl.

**Table 5:** Results of generalized linear model analysis, including significance (–: not significant, *: $p < 0.05$, **: $p < 0.001$, ***: $p < 0.0001$) of each predictor on the certainty of correct classification per model and frozen layer configuration (n$_{\text {fl}}$). CV$_{\text {R}_{\text {amp}}}$: coefficient of variation of R peak amplitude, CV$_{\text {RR}}$ : coefficient of variation of RR interval, CV$_{\text {RR}}{\times }$label: interaction between coefficient of variation of RR interval and label.

Model	Predictor	n$_{\text {{fl}}}$							
		Orig.	0	3	4	5	6	7	8
R-AF-LTM	CV$_{\text {RR}}$	**	***	***	***	***	***	***	***
	CV$_{\text {R}_{\text {amp}}}$	–	*	*	–	***	–	–	–
	Label	***	***	***	**	**	***	***	***
	CV$_{\text {RR}}{\times }$label	***	***	***	***	***	***	***	***
M-AF-LTM	CV$_{\text {RR}}$	**	**	**	**	**	–	–	–
	CV$_{\text {R}_{\text {amp}}}$	–	–	–	–	–	–	–	–
	Label	***	**	**	***	**	*	*	***
	CV$_{\text {RR}}{\times }$label	***	***	***	*	***	*	**	–
R-AF-STM	CV$_{\text {RR}}$	**	–	–	**	–	*	–	*
	CV$_{\text {R}_{\text {amp}}}$	–	–	–	–	–	–	–	–
	Label	***	*	**	**	**	*	**	–
	CV$_{\text {RR}}{\times }$label	***	**	***	***	***	***	**	*
M-AF-STM	CV$_{\text {RR}}$	**	**	*	–	*	**	*	–
	CV$_{\text {R}_{\text {amp}}}$	–	–	–	–	–	–	–	–
	Label	***	**	–	–	***	***	–	*
	CV$_{\text {RR}}{\times }$label	***	***	***	***	–	***	***	–

The application of TL using an iterative weight freezing schedule, combined with the use of an established xAI method and a systematic analysis of model-specific explanations revealed informative patterns in the decision-making of DNNs and distinguish our study from previous approaches employing TL for arrhythmia classification. While previous work has primarily evaluated TL in terms of classification performance, we conducted a comprehensive investigation of its effect on the domain specificity of model explanations. Specifically, we employed two distinct source tasks from two complementary feature domains, rhythmic and morphological, and systematically evaluated main-task and cross-task pairings between models and source domains across multiple fl configurations. This design enabled a controlled comparison of how source task selection and the degree of knowledge retention influence both classification outcomes and the spatial distribution of post-hoc explanations. Although an optimal fine-tuning schedule in terms of combined performance and explanation alignment could not be derived, the results suggested that TL can direct the focus of CNNs toward clinically relevant ECG features. In particular, the R-AF-LTM with increasing fl progressively concentrated its explanations on the rhythmic spacing of QRS complexes, while the M-AF-STM with more fl shifted its focus toward P waves in non-AF and fibrillatory activity in AF signals. These findings suggest that inductive TL may provide a viable strategy for producing explanations that better align with clinically meaningful ECG regions.

More broadly, this work can be viewed within the emerging vision of human-centered and context-aware healthcare AI, in which clinical decision-support systems should not only achieve high predictive performance, but also provide transparent and clinically meaningful information to healthcare professionals. Tan et al.^58^ emphasized that next-generation healthcare systems should integrate biological, lifestyle, environmental, and real-time contextual data while prioritizing human-centered care and trust. In this context, the proposed ECG-based TL pipeline represents a focused step toward more transparent cardiovascular AI by encouraging explanation patterns that align with clinically meaningful ECG features. The ability to guide model explanations toward diagnostically relevant features through source task selection and weight freezing may support future efforts toward the clinical validation of DL-based diagnostic systems, where interpretability is increasingly important. However, the present study evaluated fine-tuning and explanation behavior on a single target framework and did not assess whether the observed explanation patterns generalize across independent cohorts, acquisition devices, institutions, or patient populations. Therefore, conclusions regarding clinical applicability should be interpreted cautiously. Moreover, DTD relevance attributions highlight signal regions associated with model predictions and should not be interpreted as direct evidence of causal feature usage by the network. The proposed framework is not inherently restricted to AF classification and could in principle be extended to other cardiac conditions or biosignal modalities where complementary feature domains can be identified. Future work should focus on deriving principled strategies for fl selection that balance classification performance and explanation alignment, as well as on validating the robustness of the observed explanation patterns across larger and more diverse patient cohorts.

## Methods

### Data material and preprocessing

We created three task-specific datasets from publicly available databases to train and validate xECGArch on the pre-training and fine-tuning tasks. Einthoven lead II was exclusively extracted from each dataset as it is well suited for P wave analysis and AF detection^59^.

For the detection of P waves, we created the morphology dataset using 12 30-minute ECGs from the MIT-BIH Arrhythmia Database (MITDB)^51,52^, 200 10-second ECGs from the Lobachevsky University Electrocardiography Database^53^ and 50 2-minute ECGs from the Brno University of Technology ECG Signal Database (BUTDB)^54^. The BUTDB and MITDB were both annotated by two ECG experts with more than 5 years of experience, while the Lobachevsky annotations were performed by cardiologists. Each recording was resampled to 500 Hertz (Hz), divided into 10-second segments and filtered with a two-stage median filter^60^ and smoothed with a Tukey window ($\alpha$ = 0.06), followed by signal normalization. A relative inaccuracy of the P wave annotations was observed, therefore two open-source annotators^61,62^ were used to detect the local maxima along each signal and the detected peaks were compared with the original P wave annotations. P wave peaks found by both annotators were included. After preprocessing, a total of 2,716 recordings were included.

For SDRR prediction, the rhythm dataset included recordings from the following 12-lead ECG databases: PTB-XL^46,47^ (6,201 recordings), Georgia-12-Lead^48^ (993 recordings), China Physiological Signal Challenge 2018^49^ (2,871 recordings) and Chapman-Shaoxing^50^ (8,982 recordings). Of the total 19,046 recordings, 6,062 are classified as normal sinus rhythm, sinus bradycardia or sinus tachycardia, 1,351 as AF and 11,634 as other pathologies. A Butterworth high-pass filter^63^ with cutoff frequency at 0.3 Hz and order 4 was applied to the signals, followed by Tukey windowing ($\alpha$ = 0.06) and normalization. R peak annotations were created using 6 open-source QRS detectors^61,62,64–67^. An R peak annotation was accepted if a peak was found within a window of 0.1 seconds by at least two detectors and placed at the midpoint between the detections. A total of 51 incorrectly annotated samples were removed.

The third dataset, the xECGArch dataset, was created by Göttling et al.^15^ and contained a total of 9,854 10-s recordings from the same databases of the rhythm dataset. Half of the recordings are classified as non-AF and the rest as AF. The preprocessing was performed as described by Göttling et al.^15^.

Mirror padding was applied to each signal using a padding size congruent with the receptive field of each model (5,000 samples when training the LTM, and 300 samples when training the STM). Table 6 summarizes the characteristics of each dataset, including the distribution of sex, age, HR and label. The HR was derived using the LTM employed as a QRS detector^56^.

### Model training and validation

xECGArch comprises two parallel 1D CNNs, named STM and LTM. Both networks include nine convolutional blocks (convolution, batch normalization, and ReLU activation), followed by global average pooling (GAP) and a fully connected layer with two nodes and softmax activation. The two networks differ in their receptive field size, equal to 5,000 samples or 10 s in the LTM and 300 samples or 0.6 s in the STM. This difference in parameterization makes the STM more sensitive to short-term changes within beats, while the LTM considers the whole signal duration at once.

During pre-training, the LTM and STM were assigned to their primary tasks and then to their respective cross-tasks. In each experiment, the model was trained in a five-fold cross-validation grid search with the hyperparameters lr $\{0.0001, 0.001\}$ and bs $\{4, 8, 16\}$.

For the rhythmic task, the networks were modified into the R-LTM and R-STM by replacing the last layer with a single-node fully connected layer with linear activation. 15% of the rhythm dataset samples were held out for testing and the 5 folds were obtained by randomly holding 20% of the training data for validation. Huber loss^68^ was optimized with Adam^69^ and the models were trained on each fold for a total of 60 epochs, reducing the lr on plateau by a factor of 0.1.

For the morphological task, the networks were modified into the M-STM and M-LTM by removing the global average pooling and the output layer from the original models. A $10_{th}$ convolutional layer with a single output feature map and kernel size 1, batch normalization and sigmoid activation were added. The model outputs a probability for each data point of being at the peak of a P wave. A weighted binary cross-entropy loss using class weights of 0.3 for class 0 and 0.7 for class 1 was optimized with Adam.

**Table 6:** Dataset description for pre-training and fine-tuning tasks. *AF*: atrial fibrillation, *bpm*: beats per minute, *f*: female, HR: heart rate, *m*: male, *N*: normal sinus rhythm, n: number of samples, *O*: other pathology, *SD*: standard deviation, SDRR: standard deviation of RR intervals.

Set	Class	n	Sex [%]	Age	HR [*bpm*]	Label [%]
			*f/m*	*mean* ± *SD*	*mean* ± *SD*	*AF/N/O*
Pre-training (SDRR prediction)						
Training	-	16,189	45.7/54.3	$64.3 \pm 17.4$	$90.3 \pm 26.0$	7.1/31.9/61.0
Test	-	2,858	45.5/54.4	$64.4 \pm 17.6$	$91.3 \pm 27.1$	7.1/31.4/61.5
**Total**	-	19,047	45.7/54.3	$64.3 \pm 17.4$	$90.4 \pm 26.2$	7.1/31.8/61.1
Pre-training (P wave detection)						
Training	-	2,501	31.1/68.9	$53.0 \pm 17.9$	$75.8 \pm 23.1$	3.3/69.5/27.2
Test	-	215	59.0/41.0	$37.8 \pm 17.1$	$84.5 \pm 12.0$	0.5/73.0/26.5
**Total**	-	2,716	37.0/63.0	$49.5 \pm 18.8$	$76.7 \pm 22.4$	3.3/69.5/27.2
Fine-tuning (AF detection)						
Training	AF	4,420	42.3/57.7	$71.9 \pm 11.8$		
	n-AF	4,448	45.9/54.1	$60.7 \pm 16.7$		
	**Total**	8,868	44.1/55.9	$66.3 \pm 15.5$	$90.7 \pm 27.5$	49.8/5.1/45.1
Test	AF	507	42.2/57.8	$71.6 \pm 12.3$		
	n-AF	479	44.1/55.9	$60.6 \pm 17.5$		
	**Total**	986	43.1/56.9	$66.3 \pm 16.0$	$90.6 \pm 26.6$	51.4/4.1/44.5
**Total**	AF	4,927	42.3/57.7	$71.9 \pm 11.9$		
	n-AF	4,927	45.7/54.3	$60.7 \pm 16.8$		
	**Total**	9,854	44.0/56.0	$66.3 \pm 15.6$	$90.7 \pm 27.4$	50.0/5.0/45.0

The training set was obtained from 44 subjects originally belonging to the BUTDB, 11 from the MITDB and 160 from the Lobachevsky, for a total of 2,621 recordings. The remaining 49 subjects (267 recordings) were assigned to the test set. In each fold, the validation set contained recordings from 2 subjects of the MITDB, 9 subjects of the BUTDB and 26 subjects of the Lobachevsky. The model was trained for up to 80 epochs on each fold, reducing the initial lr of 0.0001 by a factor of 0.5 if no improvement in F1 score was observed for 10 epochs.

During fine-tuning, each modified version of the LTM and STM was initialized with the pre-trained weights. The models were fine-tuned on AF detection without freezing any layer and by freezing the first 3 to 8 convolutional layers, resulting in 7 fine-tuning configurations per model. The xECGArch dataset was split into 80% training and 20% test. Each model was trained for up to 80 epochs monitoring $F1_{cv} = F1_{mean} - F1_{std}$ obtained over the folds. Sparse categorical cross-entropy loss was optimized with Adam and the lr was reduced by a factor of 0.1 if the validation accuracy did not improve after 7 epochs.

In all experimental phases, dataset splits were performed at the subject level, ensuring that recordings from the same patient were exclusively assigned to either the training, validation, or test set, thereby preventing data leakage. Because the rhythm pre-training dataset and the xECGArch dataset were derived from the same four public ECG databases, we explicitly assessed potential overlap between source-task and target-task data. No patient, ECG recording, or segmented ECG sample from the rhythm pre-training dataset was present in the xECGArch test split used for final evaluation. Therefore, all reported classification performance and DTD-based explanation analyses were obtained on data that were independent from the pre-training dataset. Partial overlap existed only between the rhythm pre-training dataset and the xECGArch fine-tuning training split, amounting to 8.5% of recordings. Since this overlap was restricted to the training partitions and did not involve the independent test set, it could not affect the reported held-out performance metrics or explanation analyses.

### Statistical analysis of model explanations

Göttling et al.^15^ previously validated 13 xAI methods to generate post-hoc explanations for xECGArch, and DTD was identified as the most suitable method. Therefore, DTD with the max activation rule and bounded output between 0 and 1 was applied to extract model explanations. Using DTD, the relevance was propagated from the last convolutional layer and heatmaps were derived for each test signal of the xECGArch dataset. The heatmaps were scaled on a per-recording basis to [0,1], obtaining the rR of each signal. Mean rR per interval and recording was calculated as done by Hammer et al.^17^ and leveraging iterative two-dimensional signal warping (i2DSW)^70,71^ to extract the fiducial points. The intervals relevant for the evaluation of the explanations by the rhythm pre-trained models included the interval from Q peak to R peak (Q), the R peak ± 1 sample (R), the interval from R peak to S peak (S), and all regions outside the QRS complex (nQRS). For the morphology pre-trained models, the intervals included the P wave, the F wave, the TQ interval with F waves (F$_{\text {TQ}}$) and without F waves (nF$_{\text {TQ}}$), and all regions outside the P and F waves (nP/nF). A 2-factor ANOVA was conducted to investigate the effect of intervals and number of fl on the mean rR, averaged per interval category and ECG recording, followed by post-hoc analyses using Student’s *t*-tests with Tukey-Kramer alpha-error correction and Cohen’s *d*^55^ for effect sizes. As a robustness analysis, additional linear mixed-effects models were fitted using recording as a random intercept. Interval-level models were fitted to the target-domain intervals only, R peaks for rhythm pre-trained models and P and F wave regions for morphology pre-trained models, to assess the effect of fl configuration on domain-specific relevance. Furthermore, recording-level models including all interval categories were fitted to assess the effects of interval, fl configuration, and their interaction while accounting for repeated interval measurements within recordings.

## Supplementary Information

Supplementary Information.

## Data Availability

The datasets used in this study are publicly available: the MIT-BIH Arrhythmia Database P-Wave Annotations: https://doi.org/10.13026/C2108F, the Lobachevsky University Electrocardiography Database: https://doi.org/10.13026/eegm-h675, the Brno University of Technology ECG Quality Database: https://doi.org/10.13026/kah4-0w24, the PTB-XL electrocardiography dataset: https://doi.org/10.13026/kfzx-aw45, the Chapman-Shaoxing 12-lead ECG database: https://doi.org/10.13026/wgex-er52, the Georgia 12-lead ECG Challenge database: https://doi.org/10.13026/dvyd-kd57, and the China Physiological Signal Challenge 2018 dataset: http://2018.icbeb.org/Challenge.html. The relevance data will be made available upon publication at https://zenodo.org/records/19555343. Additional data supporting the findings of this study are available from the corresponding author upon reasonable request.

## Code availability

The MATLAB scripts used for statistical analysis and ECG relevance visualization, based on the relevance data and segment boundaries, will be made publicly available upon publication at https://github.com/MatteoIBMT/TransferLearning_AF_Explanations.
