# Supplementary material for: Cross-domain transfer learning strategy enhances interpretability of deep learning model explanations
Source: Sci Rep. 2026 Jun 24;16:19583. doi: 10.1038/s41598-026-59076-8 (PMC13294490; doi:10.1038/s41598-026-59076-8)
Supplement: Supplementary file 1 — Supplementary Information. [file 41598_2026_59076_MOESM1_ESM.pdf]

Supplementary contents

Supplementary mixed-effects model analyses

R-AF-LTM

```
Interval-level model:
Formula: rR ~ Config + (1 | Recording)

Number of observations: 101904
Fixed effects coefficients: 8
Random effects coefficients: 984
Covariance parameters: 2

Random effects covariance parameters:
Recording intercept SD = 0.09582
Residual SD = 0.23789

ANOVA marginal tests:
Term            FStat     DF1        DF2        pValue
Config          2097.1      7      100920      <0.0001

Recording-level model:
Formula: rR ~ Interval * Config + (1 | Recording)

Number of observations: 31456
Fixed effects coefficients: 32
Random effects coefficients: 984
Covariance parameters: 2

Random effects covariance parameters:
Recording intercept SD = 0.05070
Residual SD = 0.11078

ANOVA marginal tests:
Term                    FStat     DF1      DF2       pValue
Interval                1175.3      3     30441      <0.0001
Config                   309.9      7     30440      <0.0001
Interval:Config          108.5     21     30440      <0.0001
```

R-AF-STM

```
Interval-level model:
Formula: rR ~ Config + (1 | Recording)

Number of observations: 101904
Fixed effects coefficients: 8
Random effects coefficients: 984
Covariance parameters: 2

Random effects covariance parameters:
Recording intercept SD = 0.09436
Residual SD = 0.17317

ANOVA marginal tests:
Term            FStat     DF1        DF2        pValue
Config          7308.8      7      100910      <0.0001

Recording-level model:
```

Formula: rR ~ Interval * Config + (1 | Recording)

Number of observations: 31456
Fixed effects coefficients: 32
Random effects coefficients: 984
Covariance parameters: 2

Random effects covariance parameters:
Recording intercept SD = 0.04434
Residual SD = 0.12718

ANOVA marginal tests:

Term	FStat	DF1	DF2	pValue
Interval	2677.4	3	30441	<0.0001
Config	399.8	7	30440	<0.0001
Interval:Config	381.8	21	30440	<0.0001

M-AF-STM

Interval-level model:
Formula: rR ~ Config + (1 | Recording)

Number of observations: 83000
Fixed effects coefficients: 8
Random effects coefficients: 763
Covariance parameters: 2

Random effects covariance parameters:
Recording intercept SD = 0.10928
Residual SD = 0.16104

ANOVA marginal tests:

Term	FStat	DF1	DF2	pValue
Config	2760.2	7	82230	<0.0001

Recording-level model:
Formula: rR ~ Interval * Config + (1 | Recording)

Number of observations: 20082
Fixed effects coefficients: 32
Random effects coefficients: 984
Covariance parameters: 2

Random effects covariance parameters:
Recording intercept SD = 0.04659
Residual SD = 0.09719

ANOVA marginal tests:

Term	FStat	DF1	DF2	pValue
Interval	525.9	3	19208	<0.0001
Config	731.4	7	19027	<0.0001
Interval:Config	195.1	21	19037	<0.0001

M-AF-LTM

Interval-level model:
Formula: rR ~ Config + (1 | Recording)

Number of observations: 83000
Fixed effects coefficients: 8
Random effects coefficients: 763
Covariance parameters: 2

Random effects covariance parameters:
Recording intercept SD = 0.06674
Residual SD = 0.11290

ANOVA marginal tests:

Term	FStat	DF1	DF2	pValue
Config	7620.1	7	82234	<0.0001

Recording-level model:
Formula: rR ~ Interval * Config + (1 | Recording)

Number of observations: 19982
Fixed effects coefficients: 32
Random effects coefficients: 984
Covariance parameters: 2

Random effects covariance parameters:
Recording intercept SD = 0.03617
Residual SD = 0.06999

ANOVA marginal tests:

Term	FStat	DF1	DF2	pValue
Interval	64.7	3	19101	<0.0001
Config	318.2	7	18948	<0.0001
Interval:Config	171.5	21	18948	<0.0001

Supplementary Figures

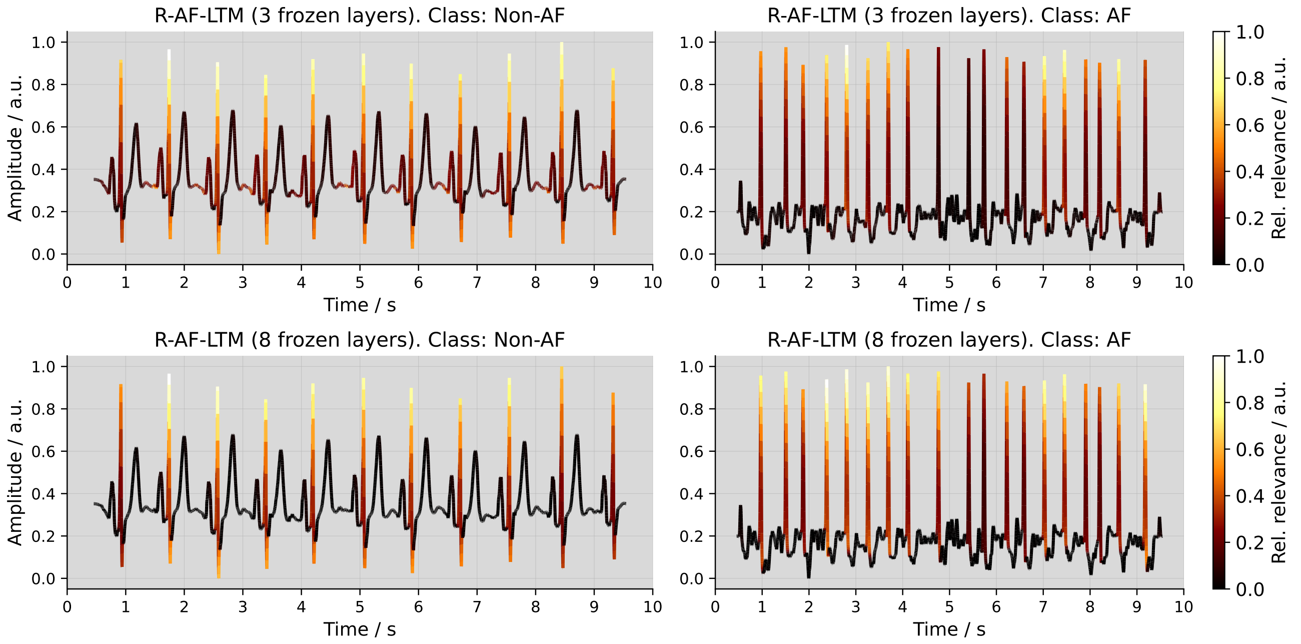

(a) ECGs, correctly classified as non-AF (patient E01362 from Georgia 12-lead database[1]) and AF (patient A1694 from China Physiological Signal Challenge 2018 database[2]) by the R-AF-LTM, configured with 3 and 8 frozen layers.

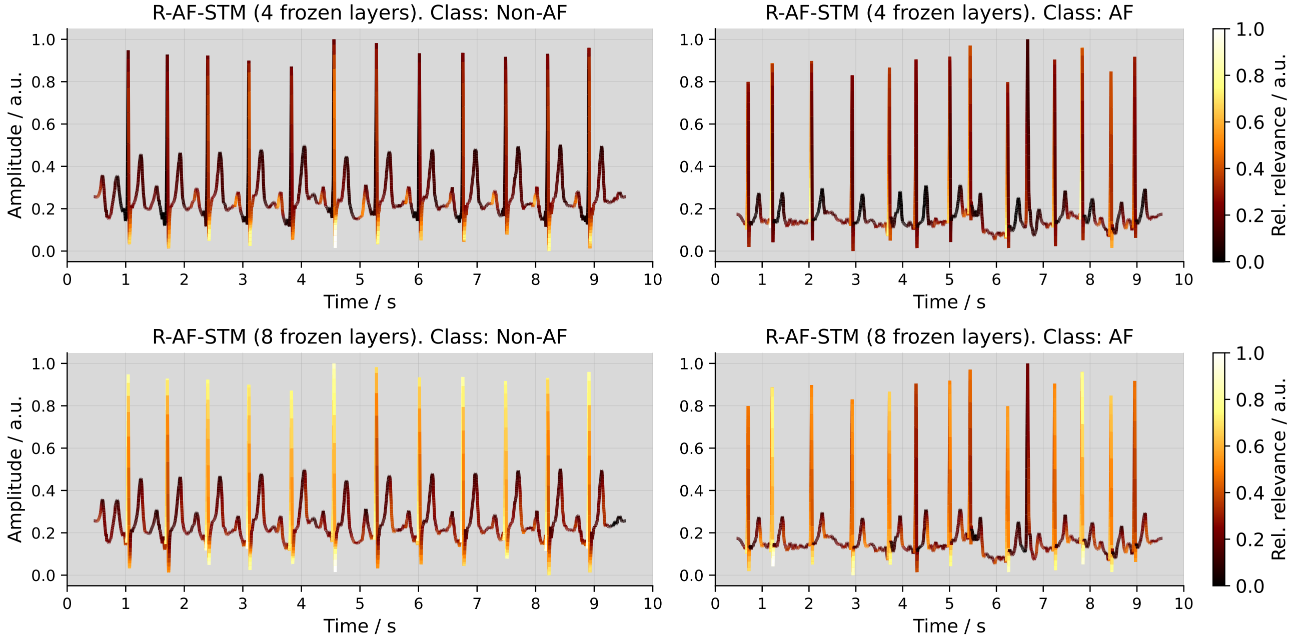

(b) ECGs, correctly classified as non-AF (patient A5478 from China Physiological Signal Challenge 2018 database[2]) and AF (patient JS01531 from Chapman-Shaoxing database[3]) by the R-AF-STM, configured with 4 and 8 frozen layers.

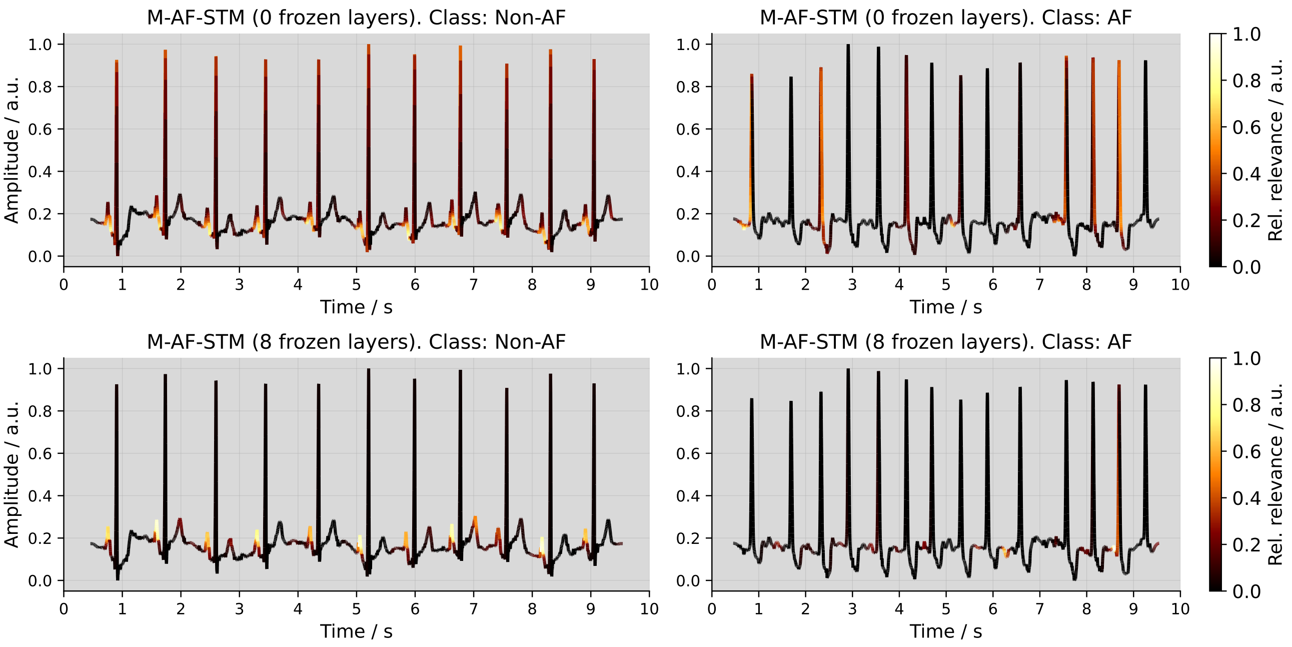

(c) ECGs, correctly classified as non-AF (patient E05624 from Georgia 12-lead database[1]) and AF (patient JS07328 from Chapman-Shaoxing database[3]) by the M-AF-STM, configured with 4 and 8 frozen layers.

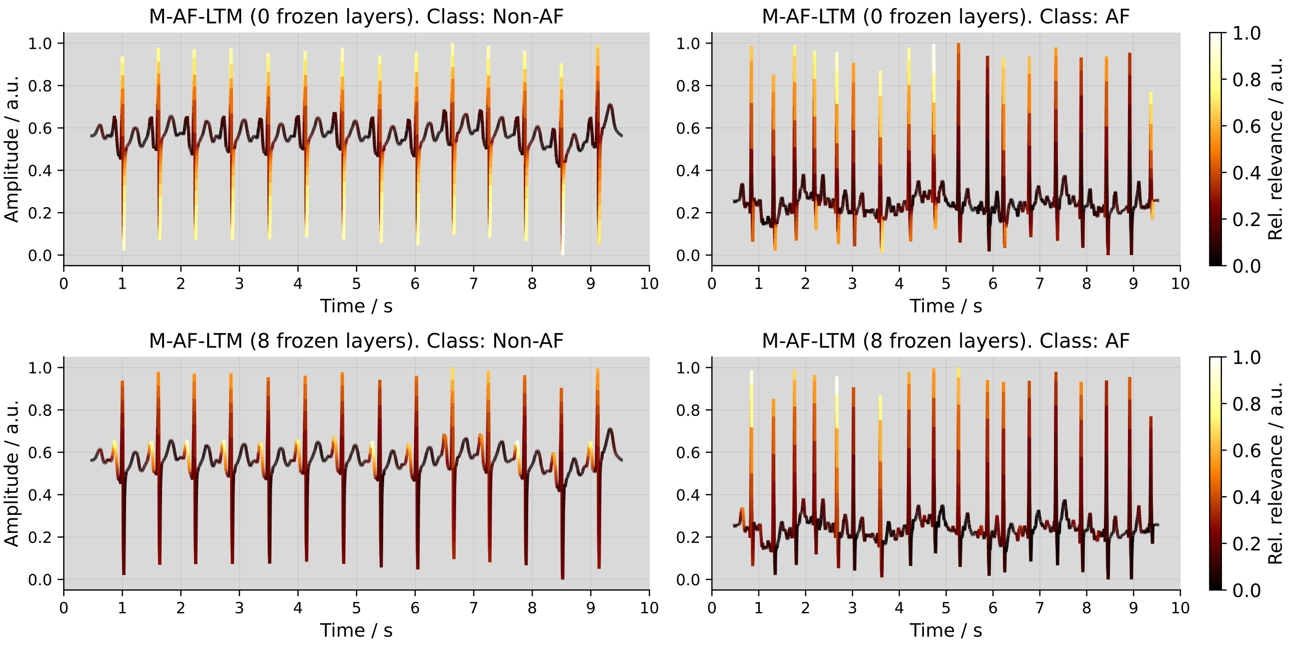

(d) ECGs, correctly classified as non-AF (patient E08471 from Georgia 12-lead database[1]) and AF (patient JS06514 from Chapman-Shaoxing database[3]) by the M-AF-LTM, configured with 4 and 8 frozen layers.

Figure S1. Explanations by deep Taylor decomposition of exemplary ECGs from the xECGArch test dataset, correctly classified as non-AF and AF by each fine-tuned model. The explanations of the best and worst performing configurations are shown.

References

1. Perez Alday, E. A. *et al.* Classification of 12-lead ECGs: the PhysioNet/Computing in Cardiology challenge 2020. *Physiol. Meas.* **41**, 124003, DOI: https://doi.org/10.1088/1361-6579/abc960 (2021).
2. Liu, F. *et al.* An open access database for evaluating the algorithms of ECG rhythm and morphology abnormal detection. *J. Med. Imaging Heal. Informatics* **8**, 1368–1373, DOI: https://doi.org/10.1166/jmihi.2018.2442 (2018).
3. Zheng, J. *et al.* A 12-lead electrocardiogram database for arrhythmia research covering more than 10,000 patients. *Sci. Data* **7**, 48, DOI: https://doi.org/10.1038/s41597-020-0386-x (2020).
